# Reality of traffic injuries attributable to preceding decreased level of consciousness

**DOI:** 10.1002/ams2.649

**Published:** 2021-05-01

**Authors:** Yuya Oda, Tomokazu Motomura, Hisashi Matsumoto

**Affiliations:** ^1^ Shock and Trauma Center Nippon Medical School Chiba Hokusoh Hospital Chiba Japan; ^2^ Department of Emergency and Critical Care Medicine Nippon Medical School Tokyo Japan; ^3^ Department of Internal Medicine Hitachiomiya City National Health Insurance Miwa Clinic Ibaraki Japan

**Keywords:** automobile driving, decreased level of consciousness, ER, traffic accident, trauma

## Abstract

**Aim:**

Although decreased level of consciousness (DLOC) while driving may lead to serious accidents involving drivers and people around them, including passengers and pedestrians, few studies have assessed traffic injuries attributable to preceding DLOC. We aimed to identify factors suggestive of a DLOC preceding traffic injury during initial examination.

**Methods:**

This study included 193 drivers who were brought to our facility during the 1‐year period from January to December 2018. The drivers were divided into those with and without DLOC for comparison and analysis. Data on age, sex, causes of DLOC, and medical history were retrospectively reviewed from medical records.

**Results:**

Of these 193 drivers, 58 (30.1%) had experienced preceding DLOC. The following factors suggested possible episodes of preceding DLOC: a single‐vehicle accident (odds ratio [OR] 3.59; 95% confidence interval [CI] 1.76–7.34; *P* < 0.001) and histories of hypertension (OR 2.64; 95% CI 1.13–6.15; *P* = 0.0248) and psychiatric disorders (OR 3.49; 95% CI 1.08–11.3; *P* = 0.0370). The causes of DLOC were endogenous diseases in 20 drivers (34.3%), dozing off episodes in 19 (32.8%), and acute alcohol intoxication in 11 (19.0%).

**Conclusion:**

Before traffic accidents, 30.1% of drivers experienced DLOC. Single‐vehicle accidents and histories of hypertension and psychiatric disorders were factors suggestive of preceding DLOC.

## Graphical abstract

30.1% of drivers had decreased level of consciousness before a traffic accident.

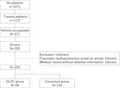

## Introduction

Decreased level of consciousness (DLOC) while driving results in drivers being unable to control their vehicles, and serious accidents involving themselves and also people around them, including passengers and pedestrians, may occur. According to a survey by the Japan Ministry of Land, Infrastructure, Transport and Tourism on commercial vehicles, annually, approximately 300 accidents are reportedly caused by health problems. Because approximately 5% of these accidents involve injury or death,^1^ countermeasures, including screening tests and the provision of guidelines for cerebrovascular disease management, have been implemented for drivers.^2^ However, few surveys on accidents attributable to health problems in all traffic accidents, including those involving private vehicles, have been conducted in Japan. Although Takuma et al. reported that accidents caused by endogenous diseases accounted for 0.4%–3.4% of traffic accidents,^3^ they failed to include accidents occurring in situations in which the drivers could not operate their vehicles normally because of dozing off episodes, acute alcohol intoxication, etc. Situations such as these may lead to serious accidents, including situations with preceding DLOC owing to endogenous diseases. It is important to conduct a fact‐finding survey on all traffic accidents, including those occurring in such situations and to suspect preceding DLOC at initial examination. This study aimed to elucidate the reality of traffic injuries involving private vehicles that are attributable to preceding DLOC owing to dozing off episodes, alcoholism, endogenous diseases, etc. and identify factors suggestive of a DLOC preceding traffic injury at initial examination.

## Methods

This retrospective observational study included drivers who had traffic injuries and were brought to the Shock and Trauma Facility, Nippon Medical School Chiba Hokusoh Hospital (hereafter, our facility) during the 1‐year period from January to December 2018. The “drivers with preceding DLOC” were defined as drivers who experienced preceding DLOC based on (i) self‐reports by interviewing injured drivers; (ii) reports from passengers or witnesses of accidents; (iii) data recorded in drive recorders; and (iv) drivers who were definitively determined to have experienced preceding DLOC by multiple physicians at our facility, including the authors, according to composite accident‐related information. Such drivers were selected based on medical records.

Drivers who might have experienced preceding DLOC but whose conditions were undeterminable because of cardiopulmonary arrests on arrival at our facility or owing to a lack of data on DLOC in medical records were excluded. The eligible injured drivers were divided into those with and without preceding DLOC (the unconscious and conscious groups) for comparison and analysis.

The study items were age, injury severity, overviews of accidents, causes of DLOC, and medical history. Severe injury was defined as Abbreviated Injury Scale score 3 or higher. The causes of DLOC were determined based on the statements of drivers and witnesses, findings during initial examination, and results of tests conducted during hospitalization. Regarding overviews of accidents, a single‐vehicle accident was defined as an accident without another party being involved.

Statistical analyses were performed using EZR (version 1.37).^4^ Categorical and continuous variables were compared using Fisher exact test and Mann–Whitney *U* test, respectively. For the multivariate analysis, a logistic regression analysis was performed to calculate odds ratios (ORs) and 95% confidence intervals (CIs). Statistical significance was set at *P* < 0.05. Continuous variables are expressed as medians and interquartile ranges (IQRs).

## Results

A total of 2,721 patients were brought to our facility during the 1‐year period. Among them, 1,172 were trauma patients including 271 vehicle occupants, of whom 205 were drivers. Twelve of the drivers were excluded, including five who died due to trauma rather than endogenous diseases and seven with unknown details. Finally, this study included 193 drivers.

**Fig. 1:**
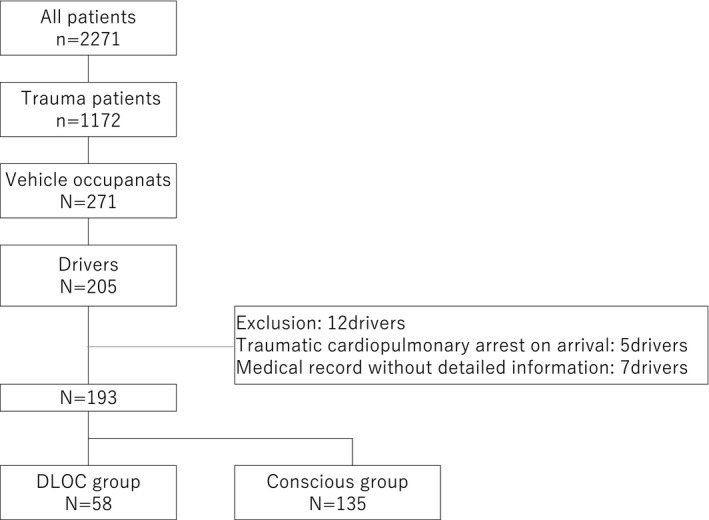
Flowchart of the study. DLOC, decreased level of consciousness.

Of these 193 drivers, 58 (30.1%) experienced preceding DLOC (Fig. 1), including 33 (56.8%), 16 (25.9%), and 3 (5.1%) who experienced preceding DLOC through interviews with them, reported by passengers or witnesses of accidents, and based on data recorded in drive recorders, respectively, and 6 (10.3%) who experienced preceding DLOC by multiple physicians based on composite information.

The median ages were 51 (IQR 29–66) and 49 (IQR 37–49) years in the unconscious and conscious groups, respectively, with no significant difference between the groups (*P* = 0.689).

There were 42 (72.4%) and 86 (63.0%) men in the unconscious and conscious groups, respectively, and there was no significant difference in the sexes (*P* = 0.319).

There were 21 (36.2%) and 44 (32.6%) severe injury patients in the unconscious and conscious groups, respectively, and there was no significant difference in severity (*P* = 0.623).

Single‐vehicle accidents were caused by 29 (50.0%) and 28 (20.7%) drivers in the unconscious and conscious groups, respectively. The proportion of single‐vehicle accidents was significantly higher in the unconscious group than in the conscious group (*P* < 0.001).

Past histories showed that 9 (15.5%) and 11 (8.2%) drivers in the unconscious and conscious groups, respectively, previously had ischemic heart diseases or arrhythmias. However, no significant differences were observed (*P* = 0.130). Furthermore, psychiatric disorders were observed in 13 (22.4%) and 7 (5.2%) drivers in the unconscious and conscious groups, respectively. The prevalence of psychiatric disorders was significantly higher in the unconscious group than in the conscious group (*P* = 0.001). Epilepsy was observed in four (6.9%) and one (0.7%) driver in the unconscious and conscious groups, respectively. The prevalence of epilepsy was significantly higher in the unconscious group than in the conscious group (*P* = 0.029). Furthermore, hypertension was observed in 20 (34.5%) and 25 (18.5%) drivers in the unconscious and conscious groups, respectively, the prevalence of which was significantly higher in the unconscious group (*P* = 0.025; Table 1).

**Table 1:** Age, sex, injury severity, single‐vehicle accidents, and medical history

	Unconscious group, *N* = 58 *N* (%)	Conscious group, *N* = 135 *N* (%)	*P* value
Age (years), median (interquartile range)	51 (29–66)	49 (37–49)	0.689
Sex (male ratio), *n* (%)	42 (72.4)	86 (63.0)	0.319
Severity (severe ratio), *n* (%)	21 (36.2)	44 (32.6)	0.623
Single‐vehicle accidents, *n* (%)	29 (50.0)	28 (20.7)	**<0.001**
With passengers, *n* (%)	14 (24.1)	43 (31.8)	0.307
Medical history, *n* (%)			
Cardiac disease	9 (15.5)	11 (8.15)	0.130
Respiratory disease	6 (10.3)	10 (7.41)	0.571
Gastrointestinal disease	6 (10.3)	13 (9.63)	1.00
Neurological disease	6 (10.3)	9 (6.67)	0.390
Renal and urologic diseases	5 (8.62)	6 (4.44)	0.311
Hemodialysis	2 (3.45)	3 (2.22)	0.638
Psychiatric disorder	13 (22.4)	7 (5.19)	**0.001**
Epilepsy	4 (6.90)	1 (0.74)	**0.029**
Hypertension	20 (34.5)	25 (18.5)	**0.025**
Diabetes mellitus	11 (19.0)	12 (8.89)	0.055

Bold type indicates a significant difference.

Furthermore, a logistic regression analysis was performed with age, sex, single‐vehicle accidents, and medical histories that were identified to have significant differences in the univariate analysis (hypertension, psychiatric disorders, and epilepsy). The risk factors for accidents following DLOC were a single‐vehicle accident (OR 3.59; 95% CI 1.76–7.34; *P* < 0.001) and histories of hypertension (OR 2.64; 95% CI 1.13–6.15; *P* = 0.0248) and psychiatric disorders (OR 3.49; 95% CI 1.08–11.3; *P* = 0.0370; Table 2).

**Table 2:** Factors influencing decreased level of consciousness

	OR	95% CI	*P* value
Sex	0.677	0.323–1.42	0.300
Age	1.00	0.979–1.02	0.963
Single‐vehicle accident	3.59	1.76–7.34	**<0.001**
Hypertension	2.64	1.13–6.15	**0.0248**
Psychiatric disorders	3.49	1.08–11.3	**0.0370**
Epilepsy	6.74	0.701–64.8	0.0985

Bold type indicates a significant difference.

95% CI, 95% confidence interval; OR, odd ratio.

Of 58 drivers in the unconscious group, three (5.2%) previously caused traffic accidents attributable to preceding DLOC.

The most common cause of DLOC was dozing off episodes in 19 drivers (32.8%), followed by acute alcohol intoxication in 11 drivers (19.0%). The most common endogenous disease was arrhythmia in six drivers (10.3%), followed by infection in five (8.6%), epilepsy in 4 (6.9%), subarachnoid hemorrhage in 2 (3.4%), and aortic dissection in 2 (3.4%; Table 3).

**Table 3:** Causes of decreased level of consciousness (N=58)

Causes of decreased level of consciousness	Number of drivers (*n*)	Proportion (%)
Dozing off	19	32.8
Acute alcohol intoxication	11	19.0
Endogenous diseases		
Arrhythmia	6	10.3
Infection	5	8.6
Epilepsy	4	6.9
Subarachnoid hemorrhage	2	3.4
Aortic dissection	2	3.4
Vagal reflex	1	1.7
Unidentified cause	8	13.8
Total	58	

No cause was identified in eight drivers, including three whose causes were not identified despite detailed examinations, including electroencephalography and Holter electrocardiography, performed during hospitalization and at the outpatient clinic, and five who were referred to another hospital and were not followed up.

Of six drivers with arrhythmias causing DLOC, one had a life‐threatening complete atrioventricular block and two had bradycardic atrial fibrillation. All six drivers exhibited arrhythmias, including premature ventricular contraction, atrial fibrillation, and atrioventricular block when they first entered the emergency department. Although one of them had noted palpitations before the DLOC, this driver did not experience palpitations after admission. Because 24‐h electrocardiography did not detect tachyarrhythmia that could cause DLOC, a loop electrocardiograph recorder was implanted in this driver.

All four drivers with epileptic seizures causing DLOC were previously diagnosed with epilepsy and were taking oral antiepileptic drugs.

Of 19 drivers with dozing off episodes causing preceding DLOC, five (26.3%) had a history of psychiatric disorders and were taking oral antipsychotic and anxiolytic drugs. Two drivers (10.1%) were taking oral antiallergic drugs (olopatadine hydrochloride and levocetirizine hydrochloride) for allergic rhinitis and two (10.1%) were taking an oral neurologic pain reliever (Pregabalin) for chronic pain.

Of 11 drivers with acute alcohol intoxication causing preceding DLOC, four (36.3%) had a history of psychiatric disorders.

## Discussion

Among drivers brought to our facility because of traffic injuries, 30.1% had injuries attributable to preceding DLOC, whereas 14.5% had injuries caused by endogenous diseases besides dozing off and acute alcohol intoxication, which was much higher than the previously reported rate of 0.4%–3.4%.^3^

Factors suggestive of DLOC preceding traffic injury at initial examination were (i) a single‐vehicle accident and histories of (ii) hypertension and (iii) psychiatric disorders.

The most common cause of preceding DLOC was dozing off episodes (32.8%). The situations wherein drivers cannot operate their vehicles normally because of dozing off episodes lead to serious accidents, similar to cases with preceding DLOC owing to endogenous diseases. Of 19 drivers with traffic injuries caused by dozing off episodes, the usage of these drugs was not shown, although 9 (47.3%) were taking oral antipsychotics, anxiolytics, and other drugs. The package inserts of antipsychotic drugs, anxiolytic drugs, antiallergic drugs (olopatadine hydrochloride and levocetirizine hydrochloride), and neurological pain relievers (pregabalin) advise users to not engage in risky machine operations, including driving. Although the Japan Ministry of Health, Labour and Welfare had issued a notification that physicians prescribing and pharmacists dispensing these drugs should explain precautions,^5^ the patients are unaware of the disclaimer about driving while using the drug. In the future, medical professionals should take further efforts to disseminate precautions to patients.

A history of psychiatric disorders was strongly associated with accidents following DLOC (OR 3.49; 95% CI 1.08–11.3). Because the oral administration of anxiolytic drugs, including benzodiazepines, increases the risk of traffic accidents,^6^, ^7^ drowsiness, which is an adverse reaction to oral antipsychotic and anxiolytic drugs, might have caused a preceding DLOC. Moreover, 4 of 11 drivers with acute alcohol intoxication causing a preceding DLOC had a history of psychiatric disorders. There may be an association between drinking and a history of psychiatric disorders.

A history of hypertension was strongly associated with accidents following DLOC (OR 2.64; 95% CI 1.13–6.15). The reason has not been clarified in this study. However, approximately 10% of patients with hypertension in Japan have moderate or severe sleep apnea syndrome.^8^ Further, patients with sleep apnea had an OR of 6.3 for having a traffic accident as compared with those without reported sleep apnea.^9^ Therefore, this study might have potentially included some drivers with hypertension and undiagnosed sleep apnea syndrome.

A single‐vehicle accident was strongly associated with accidents following DLOC (OR 3.59; 95% CI 1.76–7.34). This was due to the fact that a single‐vehicle collision accident with a stationary object is a factor caused only by the accident vehicle, including the preceding DLOC. In the case of a nonsingle accident, the factor was caused not only by the accident vehicle but also by the other vehicle.

**Fig. 2:**
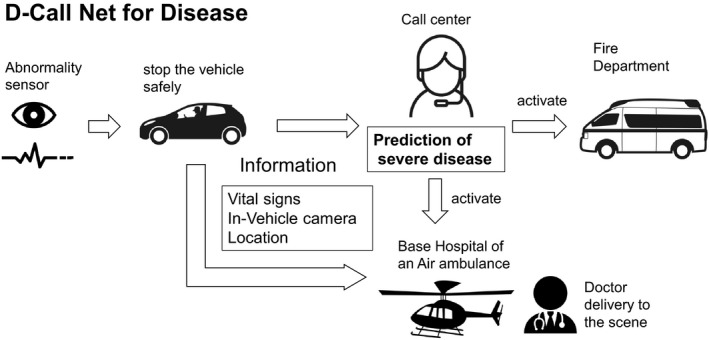
Future scenario: D‐Call Net for Disease. D‐Call Net is an advanced automatic collision notification system in Japan. Vehicle is equipped with sensors that monitors consciousness, respiratory rates, and pulse rates of occupants through seats, handles, and in‐vehicle cameras. When drivers cannot operate the vehicle normally, the system should safely stop the vehicle and automatically activate to the emergency system. The severity is predicted based on vehicle sensors and cameras. In severe predictions, call centers request the dispatch of an air ambulance. A doctor is delivered to the scene and provides early medical intervention.

A survey on accidents involving commercial drivers indicated that 36% of accidents caused by changes in physical conditions resulted in death.^10^ When the preceding DLOC or impaired consciousness owing to accidents persists, drivers are unable to summon an ambulance by themselves. Single‐vehicle accidents are likely to be caused by preceding DLOC. In such accidents that do not involve vehicles of the other party, drivers are unable to summon an ambulance; moreover, an impaired consciousness may lead to life‐threatening pathological conditions, including airway obstruction. In Japan, an advanced automatic collision notification system, called D‐Call Net, has been implemented since 2015. This system calculates the probability of death or severe injury of occupants using vehicle‐related information, including changes in speed, direction of collision, and state of the safety devices in the event of an accident and requests for dispatch of an air ambulance. This system has shortened the time between the occurrence of an accident and the request for an air ambulance by 17 min.^11^, ^12^ If endogenous diseases of occupants are also monitored and graded in severity by a private vehicle, access to a similar system may allow for early medical intervention and improve the survival rate. In the future, when drivers cannot operate the vehicle normally, the system should safely stop the vehicle and automatically activate to the emergency system including D‐Call Net (Fig. 2). It is necessary to share the reality of traffic injuries revealed by the study findings with others in the automobile industry and to advance the development of safer vehicles through collaborations between medical institutions and the industry.

This study has two methodological limitations. First, this was a single‐facility observational study conducted at the Shock and Trauma Facility. Second, it included some drivers with retrograde amnesia that hindered us from determining whether they had lost their consciousness.

Because patients were limited to suspected severe cases that were brought to our facility, we did not include any mildly injured or uninjured drivers in traffic accidents caused by DLOC. If such drivers were included, the incidence of DLOC could be higher.

The second limitation in drivers with retrograde amnesia was that we cannot determine whether they lost their consciousness unless their conditions at the time of the accident were monitored by improved automobile functions, including monitoring of occupants.

## Conclusion

This study revealed that 30.1% of drivers with traffic injury who were brought to our facility had DLOC (due to dozing off episodes, acute alcohol intoxication, endogenous diseases, etc.) before the accident. The factors suggestive of depressed level of consciousness preceding traffic injury are a single‐vehicle accident and histories of hypertension and psychiatric disorders.

## Disclosure

Approval of the research protocol: The protocol for this research project has been approved by a suitably constituted Ethics Committee of the institution and it conforms to the provisions of the Declaration of Helsinki. Committee of Nippon Medical school Chiba Hokusoh Hospital, Chiba, Japan, Approval No. 506.

Informed Consent: N/A.

Animal Studies: N/A.

Conflict of Interest: none.

## References

[1] Ministry of land, infrastructure, transport and tourism, automobile bureau: health‐related accidents and efforts to prevent health‐related accidents. 2018 edition. [cited 15 Oct 2020]. Available from: http://www.mlit.go.jp/common/001274402.pdf.

[2] Ministry of land, infrastructure, transport and tourism, automobile bureau: health management manual for drivers of commercial vehicles. [cited 15 Oct 2020]. Available from: https://www.mlit.go.jp/jidosha/anzen/03analysis/resourse/data/h26_2.pdf.

[3] Takuma K , Hori S , Koike K , *et al*. Assessment of illness as a cause of motor vehicle accidents. J. Jap. Assoc. Acute Med. 2006; 17: 177–82.

[4] Kanda Y . Investigation of the freely available easy‐to‐use software ‘EZR’ for medical statistics. Bone Marrow Transplant. 2013; 48: 452–8. 23208313 10.1038/bmt.2012.244 PMC3590441

[5] Notification of general affairs section manager, pharmaceutical and food safety bureau, safety measures section manager, may29,2013. [cited 15 Oct 2020]. Available from: https://www.mhlw.go.jp/file/06‐Seisakujouhou‐11120000‐Iyakushokuhinkyoku/130529a2.pdf.

[6] lIngebjørg G , Jørgen G.B. , Svetlana S , *et al*. Road traffic accident risk related to prescriptions of the hypnotics zopiclone, zolpidem, flunitrazepam and nitrazepam. Sleep Med. 2008; 9: 818–22. 18226959 10.1016/j.sleep.2007.11.011

[7] Orriols L , Philip P , Moore N , *et al*. Benzodiazepine‐like hypnotics and the associated risk of road traffic accidents. Clin. Pharmacol. Ther. 2011; 89: 595–601. 21368756 10.1038/clpt.2011.3

[8] Kario K . Obstructive sleep apnea syndrome and hypertension: mechanism of the linkage and 24‐h blood pressure control. Hypertens. Res. 2009; 32: 537–41. 19461649 10.1038/hr.2009.73

[9] Teran‐Santos J , Jimenez‐Gomez A , Cordero‐Guevara J . The association between sleep apnea and the risk of traffic accidents. Cooperative Group Burgos‐Santander. N. Engl. J. Med. 1999; 340: 847–51. 10080847 10.1056/NEJM199903183401104

[10] Hitosugi M , Gomei S , Okubo T , Tokudome S . Sudden illness while driving a vehicle a retrospective analysis of commercial drivers in Japan. Scand. J. Work Environ. Health 2012; 38: 84–7. 21850364 10.5271/sjweh.3189

[11] Matsumoto H , Mashiko K , Hara Y , *et al*. Dispatch of helicopter emergency medical services via advanced automatic collision notification. J. Emerg. Med. 2016; 50: 437–43. 26810021 10.1016/j.jemermed.2015.11.001

[12] Motomura T , Matsumoto H , Mashiko K , *et al*. A system that uses advanced automatic collision notification technology to dispatch doctors to traffic accidents by helicopter: the first 4 cases. J. Nippon Med. Sch. 2020; 87: 220–6. 32238730 10.1272/jnms.JNMS.2020_87-406

